# UAV Mission Planning Resistant to Weather Uncertainty

**DOI:** 10.3390/s20020515

**Published:** 2020-01-16

**Authors:** Amila Thibbotuwawa, Grzegorz Bocewicz, Grzegorz Radzki, Peter Nielsen, Zbigniew Banaszak

**Affiliations:** 1 Department of Materials and Production, Aalborg University, 9220 Aalborg, Denmark; amila@mp.aau.dk (A.T.); peter@mp.aau.dk (P.N.); 2 Faculty of Electronics and Computer Science, Koszalin University of Technology, 75-453 Koszalin, Poland; radzki.grzegorz@gmail.com (G.R.); Zbigniew.Banaszak@tu.koszalin.pl (Z.B.)

**Keywords:** Unmanned Aerial Vehicles, UAV routing and scheduling, UAV fleet mission planning

## Abstract

Fleet mission planning for Unmanned Aerial Vehicles (UAVs) is the process of creating flight plans for a specific set of objectives and typically over a time period. Due to the increasing focus on the usage of large UAVs, a key challenge is to conduct mission planning addressing changing weather conditions, collision avoidance, and energy constraints specific to these types of UAVs. This paper presents a declarative approach for solving the complex mission planning resistant to weather uncertainty. The approach has been tested on several examples, analyzing how customer satisfaction is influenced by different values of the mission parameters, such as the fleet size, travel distance, wind direction, and wind speed. Computational experiments show the results that allow assessing alternative strategies of UAV mission planning.

## 1. Introduction

Unmanned Aerial Vehicles (UAVs) are a promising maturing technology to support delivery operations due to their potential for fast, cost-effective, and more sustainable nature than traditional delivery modes such as land and sea transportation [1,2,3]. Urban Air Mobility (UAM) is an emerging solution to the challenge of congestion and pollution from transportation means increasingly found in large urban environments. UAM will reshape transportation and logistics in the future through reducing the load on land-based transportation means [3,4] and depends on the introduction of next-generation Vertical Take-Off and Landing (VTOL) vehicles capable UAVs as a mode of transport service [3]. UAM systems will introduce new innovative UAVs-related operations to the airspace across the world [3] and are expected to revolutionize the transportation infrastructure, mainly in urban areas or hard to access rural areas. To fulfill these visions requires developing technology supporting very large-scale autonomous deployment of fleets of UAVs. As a consequence, flight and navigation tasks for UAV fleets are increasingly automated to gain economies-of-scale, increase the speed of operations, and support the large-scale operations envisioned in UAM. Another aspect of UAM systems is to support monitoring, e.g., traffic conditions using UAVs to move between points of interest. Alternatively, the same methods can be used to coordinate surveillance missions where UAVs act as sensor platforms to monitor specific events such as sporting events and concerts. Enhancement in the autonomy of UAVs has changed the role of the operating personal to one of control supervision where the operator will be primarily handling the high-level mission management in contrast to low-level manual flight control. Likewise, UAV mission planning and execution is transitioning from teams of operators managing a single UAV to a single operator managing multiple UAVs [3,4]. The increasing degree of autonomy and automation has made a demand for faster and safer systems to handle complex UAV operations.

In using UAV technology to address the societal challenges through the making seamless flow of transport operations, practical constraints such as weather conditions and energy consumption make the problem highly complex and intractable [3]. Even though UAV technologies provide the more flexible transfer of goods between locations, these generate new challenges in the organization and maintenance of the planned routes and schedules [5,6]. UAVs mission planning is vital to the operations of UAV fleets and, to support autonomous operations of UAV fleets, new approaches for fleet mission planning must be developed. UAV fleet mission planning problems are by their nature an extension of the well-known Vehicle Routing Problem (VRP). However, these problems have the added complexity of combining routing and scheduling, three-dimensional operations, and non-linear fuel consumption [3,4,7]. The classical VRP is well-studied and the methods and approaches found within this domain are still very much applicable for the advancement of new technology in the area of UAV operations. However, mission planning for UAV fleets must consider a number of constraints and operating conditions rarely seen in the traditional VRP and operating conditions and limitations not found in other transportation means [3]. Some central examples of this are the constraints on UAV range that depend on weather conditions, airspace regulations and restrictions, as well as congestion in terms of collision avoidance and safety distance, and the UAV characteristics such as airspeed, maximum payload, energy capacity, physical dimensions, etc. [3,4,7,8]. In UAV mission planning, it is necessary to address weather conditions [9,10] and changes to these weather conditions can potentially strongly influence the solution strategy for the UAV mission planning, especially wind direction and speed as they directly impact energy consumption and flight characteristics [3,11]. Thus, UAV mission planning strategies must estimate energy consumption to identify the set of all reachable destinations [3,4]. As weather is by its nature uncertain, delivery services should accommodate this uncertainty through planning UAV fleet missions as a sequence of repeatedly executed UAV routes and schedules [4]. This will tend to increase delivery satisfaction by spreading risk and stabilizing deliveries.

This research presents an extension of the work presented previously [8] and presents an expanded solution approach for mission planning problems taking into account the UAV energy capacity and flight characteristics, weather uncertainty, payload weight, number of customers, customer demand, fleet size, distribution network structure, and time horizon. Special attention is devoted to the UAVs flying mission planning following the delivery strategies assuming either that each UAV travels at a constant ground speed or constant airspeed throughout the mission.

The solutions provide answers to whether a given level of customer satisfaction is achievable within a given time horizon. Similar problems have been considered in previous studies [12,13,14] and the focus of this study is on solutions that allow one to find a collision-free plan of delivery missions composed of a sequence of UAV multi-trip missions maximizing order fulfillment. This research further proposes a declarative framework that allows one to formulate a reference model for the analysis of the relationships between the structure of a given UAV-driven supply network and its resistance to weather as a result of the sequence of UAVs’ routes and schedules. The presented computational experiments and simulations provide the requirements for a solvable class of UAV-driven mission planning problems resistant to weather uncertainty and energy consumption constraints. The results fall within the scope of research previously reported in [4] and [12] and extend the work in [14].

The remainder of the article is structured as follows: Section 2 provides an overview of the literature. A motivation example introducing the considered problem is presented in Section 3. A reference model for a UAV fleet routing and scheduling problem is presented in Section 4. The problem is formulated in Section 5, which also presents a Constraint Satisfaction Problem-based method for planning UAV delivery missions. An example illustrating the approach proposed is given in Section 6. Conclusions are formulated and directions of future research are suggested in Section 7.

## 2. Literature Review

In contrast to traditional routing problems, the UAV fleet mission planning problem addresses multiple decision layers including both the fleet level where the fleet is managed in terms of task assignment and availability management and the platform level where the individual missions of the UAVs are created [3,4]. The current state of research into the area is fragmented and neglects that different types of decisions are addressed at different abstraction levels of UAV fleet mission planning [4,8,14,15]. In general, the accomplishments in the field are focused on UAV routing for transporting materials and surveillance [2,16,17,18] without considering the changing conditions in weather and non-linear energy consumption of UAVs [19].

Mission planning aims to find a sequence of points that connect the starting location to the destination location and differs from trajectory planning where the solution path is expressed in terms of the degrees of freedom of the vehicle [3,18,20]. In general vehicle routing problems, the standard objective function is typically time minimization for visiting a set number of nodes. In UAV fleet mission planning, several variants of objective functions such as reducing individual UAV costs, increasing safety in operations, reducing lead time, and increasing the load capacity of the entire system are considered [3,21,22,23,24]. Furthermore, the problem can be considered an extension of the vehicle routing and scheduling problems and belongs to the class of NP-hard problems [2,4]. The UAV fleet mission planning problem differs from the traditional time-dependent VRP as it simultaneously addresses both fleet and individual vehicle management [4].

From the literature, it is evident that the decision criteria in UAV mission planning are many and complex in nature [3,4,14]. Specifically, the decision space comprises aspects related to routing and scheduling [1,25], changing weather conditions (wind speed, wind direction, air density) [26], UAVs’ specifications [16,27], energy consumption affected by weather conditions [16], and the payload carried by the UAVs [2] as well as collision avoidance with respect to both moving objects and fixed obstacles [3,25]. Together, these elements emphasize the potential intractability of mission planning as it is highly challenging to develop models considering all these influencing aspects concurrently [3].

From the literature, one can identify that there are several aspects that necessitate treating the problem differently from traditional transportation problems. UAVs are limited by their loading capacity as well as their flight duration, which is linked to the energy capacity of the UAV [2,3,14,28]. These constraints are typically addressed in transportation problems. However, UAVs have the additional complexity that the flight duration heavily depends on the payload carried which requires these characteristics to be taken into consideration in UAV mission planning [29,30]. Weather is critical for energy consumption as it affects the travel speed of the UAV (and thus total energy consumed), and the ambient temperature affects the energy capacity [2,3,28] of batteries used in UAVs. Cold temperatures may adversely affect battery performance until the batteries warm up [2,15]. Air density at the same time provides air resistance and lift and directly affects energy consumption. Air density is a function of humidity, air pressure, and temperature [3,15]. The current state of research has yet to consider weather factors and assumes the weather has a negligible impact on performance [2,16,31,32]. Rarely has research included consideration of wind conditions’ impact on energy consumption while concurrently using that information in planning the missions of UAVs [2,33,34], with only a few contributions identifiable in current state. A number of studies have assumed constant wind speed and wind direction [33] and used linear approximations for energy consumption [2]. The technical parameters of UAVs including the UAV dimensions, battery capacity and payload limits, and the aspects of changing weather conditions, including wind speed, wind direction, and air density, all influence the search for possible UAV mission planning solutions [3,12]. As the linear approximations reported in the current state are insufficient in terms of finding acceptable energy calculations for the UAVs considered in this research study [10], non-linear models proposed are used to calculate energy consumption in relation to weather conditions [4,15].

A number of contributions have proposed to subdivide the mission area taking into account UAVs’ relative capabilities and to cluster the subsequent smaller areas to reduce the problem size [3,4,31,35,36]. Certain studies in the current state have used strategies to cluster the network to reduce the problem complexity [31,35,36,37]. Utilizing this as the foundation, this study proposes to cluster customer nodes and for each customer cluster a set of feasible weather-resistant UAV fleet schedules with routes are created. Further complexity to finding a solution is added by the challenge of collision avoidance with both fixed and flying objects [38]. Collision avoidance can be achieved by predicting potential collisions in offline planning or by reacting to collisions registered by sensors in online planning [39,40,41].

Recent studies have proposed heuristics-based decomposed solution approaches to solve the UAV fleet mission planning problem [3,4,10] and provide solutions for relatively large UAVs considering weather-dependent non-linear energy consumption. It is worth emphasizing a number of large multinational companies are today pursuing the development of UAVs on the scale addressed in this research (payload of several dozen kilograms). Among these, such companies like Airbus and Amazon stand out as significant actors. Airbus has, e.g., already commenced scale demonstrations in Singapore for cargo drones with a lift capability of 4 kg [42] and many such initiatives are underway. A number of UAVs with the Vertical Take-Off and Landing (VTOL) and lift capability described in this research already exist as functioning prototypes. For example. the Korean Aerospace Research Institute [43] has demonstrated fully functioning VTOL UAVs (e.g., TR-100) in a scale even exceeding the ones used in the example in this research (e.g., UAV with a payload of 90 kg and a flight duration of 5 h).

Furthermore, studies have formulated the mission planning problem for a fleet of UAVs as a mixed-integer non-linear programming problem and then approximated it as a mixed-integer linear programming problem and used the Gurobi environment for solving this reduced problem without considering the weather uncertainty [44]. Furthermore, in recent studies, the problem has been formulated as an extension of the VRP with time windows and solved in a constraint programming environment (IBM ILOG). This approach only enables us to provide solutions for relatively small networks [14]. Thus, there is a clear lack of methods and approaches able to provide solutions considering the resistance of mission planning to the changing weather conditions and accommodating the effects of changing weather conditions on energy consumption.

## 3. A Motivational Example

**Figure 1:**
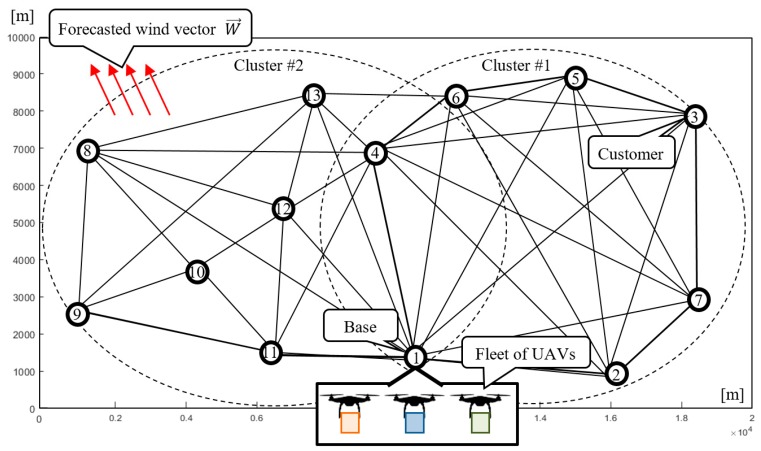
Transportation network.

Consider a company that provides air transport services using a fleet of UAVs. The transportation network covers 200 km^2^ and contains 13 nodes (one base: $N_{1}$ and 12 customers: $N_{2}-N_{13}$; see Figure 1). The fleet consists of three homogeneous UAVs with the technical parameters presented in Table 1. The horizon time and goods delivery demand of individual customers is known in advance and the goods are transported under any weather conditions where the UAVs are capable to operate. In that context, the problem under consideration can be reduced to answering the following question: *Is the available UAV fleet able to guarantee the delivery of the required quantity of goods using the given transport network within the assumed time horizon under the forecasted weather conditions?*

In other words, what one is striving to identify is a proactive flight mission plan (UAV routes and schedules) that will allow the particular fleet of UAVs, flying under given weather conditions, to deliver the required quantity of goods to customers.

**Figure 2:**
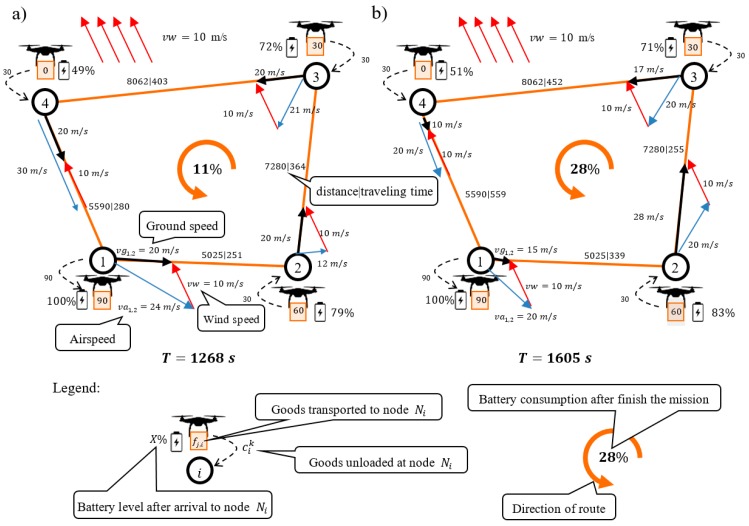
Strategies for delivering goods following a constant ground speed (**a**) and a constant airspeed (**b**).

Two main strategies for delivering goods under changing weather conditions have been proposed in the literature [14,45,46]. Their principles are illustrated in Figure 2. The first strategy (Figure 2a) assumes that a drone travels at a constant ground speed of $vg_{i,j}=20\;m/s$. In addition, it is assumed that the drone moves along route $\pi_{1}=\left(N_{1},N_{2},N_{3},N_{4},N_{1}\right)$ and carries 90 kg of goods. To be able to maintain the ground speed for each route segment under the given weather conditions ($vw_{\;}=10\;m/s$), the UAV must generate the proper airspeed (vector $\vec{va_{i,j}}$) to compensate for changes in wind direction and speed.

This results in variable energy consumption, which depends on drone speed $\vec{va_{i,j}}$ and the weight of the freight $f_{i,j}$ transported by the drone. Power $P_{i,j}$, which defines the amount of energy consumed along segment (i,j) is described by the formula below [16]:

(1) $$P_{i,j}=\frac{1}{2}C_{D}AD{\left(va_{i,j}\right)}^{3}+\frac{{\left(ep+f_{i,j}\right)}^{2}}{Db^{2}va_{i,j}},$$

where $C_{D}$, A, D, b, and ep are, respectively, the following constant parameters: drag coefficient, front surface of UAV, air density, UAV width, and UAV weight. The parameters $f_{i,j}$ and $va_{i,j}$ are the weight of the payload transported along segment  (i,j) and airspeed, respectively.

Airspeed vector $\vec{va_{i,j}}$ should compensate for the effects of the wind in such a manner that the drone can move between nodes at the speed 20 m/s. The value of parameter $va_{i,j}=\left|\vec{va_{i,j}}\right|$ is determined from the following relationship:

(2) $$va_{i,j}=\sqrt{{\left(vg_{i,j}cos\vartheta_{i,j}-vw_{\;}cos\theta \right)}^{2}+{\left(vg_{i,j}sin\vartheta_{i,j}-vw_{\;}sin\theta \right)}^{2}},$$

where $\vartheta_{i,j}$ is the angle of inclination of the ground speed vector $\vec{vg_{i,j}}$ and θ is the angle of inclination of the wind speed vector $\vec{vw_{\;}}$.

The approach presented graphically in Figure 2a guarantees a constant ground speed and hence a constant flight time along a specific route (T=1268 s). However, to maintain this speed, it is necessary to continuously adjust the flight to the current weather conditions (energy consumption varies depending on weather conditions, in particular the strength and wind direction). For this complex strategy, the energy consumption associated with delivering goods along route $\pi_{1}$ is E=89% (battery capacity CAP = 8000 kJ).

The approach illustrated in Figure 2b, in turn, assumes that airspeed is constant throughout the mission ($va_{i,j}=20\;m/s$). This results in different ground speeds ($vg_{i,j}$) for different segments of the route and a different total flight time (T=1605 s). When the airspeed is constant, power $P_{i,j}$ (1) for each route segment is independent of weather conditions ($va_{i,j}$ is constant). The energy consumption is then dependent on flight time $t_{i,j}$ and the weight of the freight $f_{i,j}$. With this strategy, to fly the entire route $\pi_{1}$, a drone has to use up E=72% of all (pre-stored) energy.

As is easily seen, the second strategy ensures a lower energy consumption at the expense of longer flight time, and it is this strategy that is widely used in flight mission planning [3,26,45,46]. A characteristic feature of the first strategy is that flight time remains constant independent of weather conditions. This feature is particularly important in situations where goods must be transported within specified time windows and/or when they are to be delivered to customers just-in-time. There are few contributions [20,24] for UAV mission planning that consider this type of delivery strategy.

**Figure 3:**
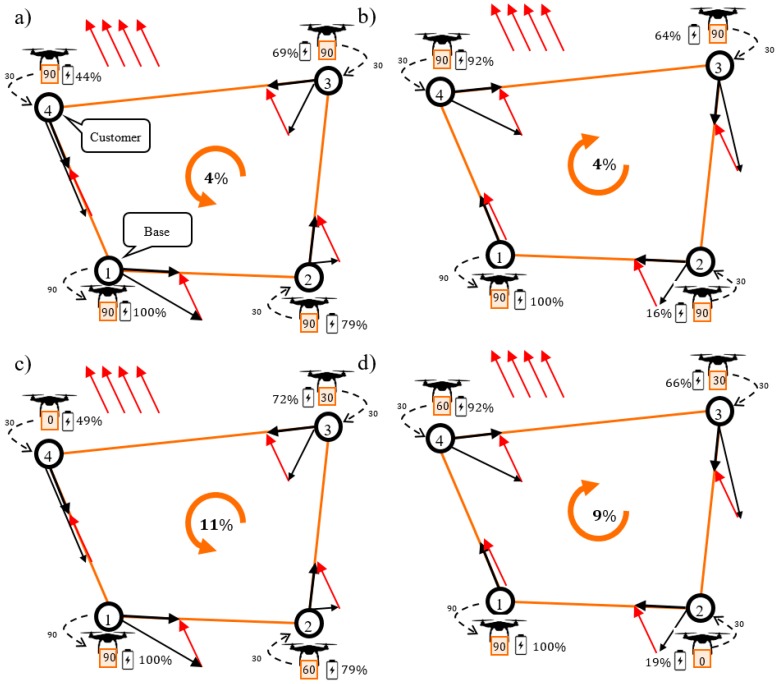
Energy consumption under a constant Unmanned Aerial Vehicles (UAV) weight (**a**,**b**), and under a variable UAV weight (**c**,**d**).

It is also worth emphasizing that most of the models encountered in the literature assume that the weight of a drone does not change during flight [16,18]. Figure 3a,b presents such a situation in which the total weight of the UAV remains unchanged during flight along route $\pi_{1}$ where energy consumption is E=96%. Figure 3c,d illustrates situations in which the weight of a UAV changes as the cargo is successively unloaded at sequential delivery points (the UAV delivers 30 kg of goods each to nodes $N_{2}$, $N_{3}$, $N_{4}$). It should be emphasized that, in this case, the energy consumption depends on the direction of flight and is E=89% when the drone flies counterclockwise and E=81%  when the drone flies clockwise.

This example shows that models in which additional features, such as the variability of UAV weight, have been taken into account can be used to generate routes (and route directions) with a lower energy consumption than the models proposed in the current state [34,37]. The introduction of new features, however, involves the need to take into account additional decision variables, which significantly increases the computational complexity of the problem.

In the next section, we present a declarative model for UAVs flying missions planning following the above-mentioned strategies assuming either constant ground speed or constant airspeed. The variability of UAVs’ weight, e.g., weight reduction during travel is also taken into account.

## 4. Modeling

### 4.1. Assumptions

**Figure 4:**
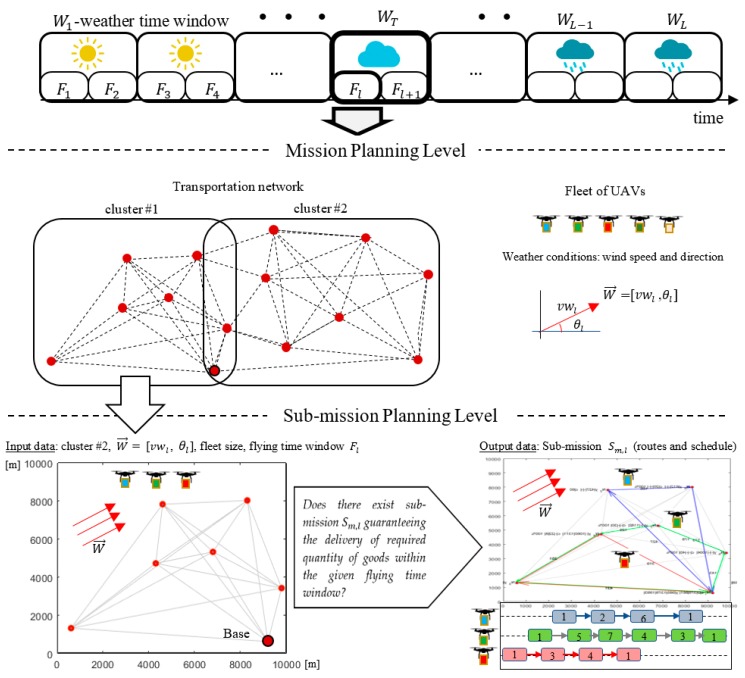
Illustration of mission planning approach.

The concept of the considered approach is presented in Figure 4. Given is a set of customers located at different points of a transportation network that are to be serviced by a fleet of UAVs during a specified time horizon, under changing weather. In this context, the following assumptions are taken into account:

- The weather forecast is known in advance with sufficient accuracy to specify the so-called weather time windows $W_{l}$.
- The weather time windows can be subdivided into flying time windows $\;F_{l}$.
- The weather (which is known in advance) is specified by vector $\vec{W_{l}}=\left[vw_{l},\theta_{l}\right]$ where $vw_{l}^{\;}$ is the wind speed and $\theta_{l}$ is the direction of wind for each $F_{l}$. Vector $\vec{W_{l}}$ is constant for a given weather time window.
- Every route traveled starts and terminates within a given flying time window.
- All UAVs are charged to their full energy capacity before the start of a flying time window, and a UAV can only fly once during a flying time window.
- The same kind of cargo is delivered to different customers in different amounts (kg).
- The weight of a UAV is decreased as the cargo is successively unloaded at customers located along its route.
- The network consists of customer locations (delivery points) and flying corridors.

The goal is to fulfill all customer demands, such that each customer is at a required service level before the end of the time horizon, and all constraints related to energy limits and congestion avoidance are satisfied. The proposed approach assumes that the process of finding solutions takes place at two levels: Mission and Sub-Mission Planning (see Figure 1). At the Mission Planning Level, the transportation network is divided into a set of clusters (covering the base and several customers) for which the size of the UAV fleet is determined. At the Sub-Mission Planning Level, the UAV sub-missions (specified by UAV routes and schedules) are calculated for each cluster. The UAV sub-missions may be calculated according to one of the following strategies:

- Strategy 1—which assumes that a UAV travels at a constant ground speed. The airspeed must compensate adverse changes in wind direction and speed.
- Strategy 2—which assumes that the UAV airspeed is constant throughout the mission. The ground speed is different for different segments and depends of the wind parameters specified by $\vec{W_{l}}$.

It is assumed that there exists a sequence of sub-missions that fulfills all customer demands within the given time horizon.

### 4.2. Declarative Model

The mathematical formulation of the model dedicated to the Sub-Mission Planning Level employs the following parameters, variables, sets, and constraints:
**Parameters***Network*G=(N,E)graph of a transportation network: N={1…n} is a set of nodes, E={{i, j}| i, j ∈N,i ≠ j} is a set of edges$CL_{m,l}=\left(N_{m,l},E_{m,l}\right)$subgraph of G representing the *m*th cluster in the *l*th flying time window: $N_{m,l}\subseteq N$ and $E_{m,l}\subseteq E$
$z_{i}$demand at node i ∈ N, $z_{1}=0$
$pr_{i}$priority of the node i ∈ N, $pr_{1}=0$$d_{i,j}$travel distance from node i to node j$t_{i,j}$travel time from node i to node jwtime spent on take-off and landing of a UAVtstime interval at which UAVs can take off from the base$b_{\left\{i,j\right\};\{\alpha ,\beta \}}^{\;}$binary variable corresponding to crossed edges
$b_{\left\{i,j\right\};\{\alpha ,\beta \}}^{\;}=\{\begin{array}{cc} 1 & \mathrm{when}\;\mathrm{edges}\;\{i,j\}\;\mathrm{and}\;\{\alpha ,\beta \}\;\mathrm{are}\;\mathrm{crossed}\\ 0 & \mathrm{otherwise}. \end{array}$*UAV Technical Parameters**K*size of the fleet of UAVs*Q*maximum loading capacity of a UAV$C_{D}$aerodynamic drag coefficient of a UAV*A*front facing area of a UAV*ep*empty weight of a UAV*D*air density*b*width of a UAVCAPmaximum energy capacity of a UAV*Environmental Parameters*Htime horizon $H=\left[0,t_{max}\right]$$W_{T}$weather time window T: $W_{T}=\left[WS_{T},WE_{T}\right]$, $WS_{T}$/$WE_{T}$ is a start/end time of $W_{T}$$F_{l}$flying time window l*:*
$F_{l}=\left[FS_{l},FE_{l}\right]$, $FS_{l}$/$FE_{l}$ is a start time of $F_{l}$$vw_{l}$wind speed in the *l*th flying time window$\theta_{l}$wind direction in the *l*th flying time window$va_{i,j}^{l}$airspeed of a UAV traveling from node i to node j in the *l*th flying time window$\varphi_{i,j}$heading angle, angle of the airspeed vector when the UAV travels from node i to node j$vg_{i,j}^{l}$ground speed of a UAV travelling from node i to node j in the *l*th flying time window$\vartheta_{i,j}$course angle, angle of the ground speed vector when the UAV travels from node i to node j**Decision Variables**$x_{i,j}^{k}$binary variable used to indicate if the *k*th UAV travels from node i to node j
$x_{i,j}^{k}=\{\begin{array}{cc} 1 & \mathrm{if}\;k\mathrm{th}\;\mathrm{UAV}\;\mathrm{travels}\;\mathrm{from}\;\mathrm{node}\;i\;\mathrm{to}\;\mathrm{node}\;j\\ 0 & \mathrm{otherwise}. \end{array}$$y_{i}^{k}$time at which the *k*th UAV arrives at node i$c_{i}^{k}$weight of freight delivered to node i by the *k*th UAV$f_{i,j}^{k}$weight of freight carried from node i to node j by the *k*th UAV$P_{i,j}^{k}$energy per unit of time, consumed by *k*th UAV during a flight from node i to node j$s^{k}$take-off time of the *k*th UAV$cp_{i}$total weight of freight delivered to node i$\pi_{m,l}^{k}$route of the *k*th UAV in the *m*th cluster in the *l*th flying time window $\pi_{m,l}^{k}=\left(v_{1},\ldots ,v_{i},v_{i+1},\ldots ,v_{\mu }\right)$, $v_{i}\in N_{m,l}$, $x_{v_{i},v_{i+1}}^{k}=1$**Sets**$Y_{\;}^{k}$set of times $y_{i}^{k}$—schedule of the *k*th UAVYfamily of $Y_{\;}^{k}$—schedule of UAV fleet$C_{\;}^{k}$set of $c_{i}^{k}$—payload weight delivered by the *k*th UAVCfamily of $C_{\;}^{k}$Πset of UAV routes $\pi_{m,l}^{k}$$S_{m,l}$sub-mission in the *m*th cluster in the *l*th flying time window $S_{m,l}=\left(\Pi ,Y,C\right)$

**Constraints**

Routes. Relationships between the variables describing drone take-off times/mission start times and task order:

(3) $$s^{k}\geq 0,k=1\ldots K$$

(4) $$\left(k\neq q\right)\Rightarrow \left(\left|s^{k}-s^{q}\right|\geq TS\right),k,q=1\ldots K$$

(5) $$\sum_{j=1}^{n}x_{1,j}^{k}=1,\;k=1\ldots K$$

(6) $$\left(x_{1,j}^{k}=1\right)\Rightarrow \left(y_{j}^{k}=s^{k}+t_{1,j}\right),j=1\ldots n;\;k=1\ldots K$$

(7) $$\left(k\neq q\;\wedge y_{i}^{k}\neq 0\;\wedge y_{i}^{q}\neq 0\right)\Rightarrow \left(\left|y_{i}^{k}-y_{i}^{k}\right|\geq w\right),i=1\ldots n;\;k,q=1\ldots K$$

(8) $$\left(x_{i,j}^{k}=1\right)\Rightarrow \left(y_{j}^{k}=y_{i}^{k}+t_{i,j}+w\right),\;j=1\ldots n;\;i=2\ldots n;\;k=1\ldots K$$

(9) $$y_{i}^{k}\geq 0,\;i=1\ldots n;\;k=1\ldots K$$

(10) $$\sum_{j=1}^{n}x_{i,j}^{k}=\;\sum_{j=1}^{n}x_{j,i}^{k},\;i=1\ldots n;\;k=1\ldots K$$

(11) $$y_{i}^{k}\leq H\times \sum_{j=1}^{n}x_{i,j}^{k},\;i=1\ldots n;\;k=1\ldots K$$

(12) $$x_{i,i}^{k}=0,\;i=1\ldots n;\;k=1\ldots K$$

Collision avoidance. Intersecting edges $\left(b_{\left\{i,j\right\};\left\{a,b\right\}}=\;1\right)$ cannot be occupied by more than one UAV at the same time $\left(x_{i,j}^{k}=1,\;x_{i,j}^{q}=1\right)$.

(13) $$\left(block_{\left\{i,j\right\}\left\{a,b\right\}}\wedge \;x_{i,j}^{k}=1\;\wedge x_{a,b}^{q}=1\right)\Rightarrow \left(y_{b}^{q}\leq y_{j}^{k}-t_{i,j}\right)\vee \left(y_{j}^{k}\leq y_{b}^{q}-t_{a,b}\right)$$

i,j=1…n; k,q=1…K; k≠q

Delivery of freight. Relationships between the variables describing the amount of freight delivered to nodes by UAVs and the demand for goods at a given node:

(14) $$c_{i}^{k}\geq 0,\;i=1\ldots n;\;k=1\ldots K$$

(15) $$c_{i}^{k}\leq Q\times \sum_{j=1}^{n}x_{i,j}^{k},\;i=1\ldots n;\;k=1\ldots K$$

(16) $$\sum_{i=1}^{n}c_{i}^{k}\leq Q,\;k=1\ldots K$$

(17) $$\left(x_{i,j}^{k}=1\right)\Rightarrow c_{j}^{k}\geq 1,\;k=1\ldots K;\;i=1\ldots n;\;j=2\ldots n$$

(18) $$\sum_{k=1}^{K}c_{i}^{k}=cp_{i},\;i=1\ldots n$$

(19) $$cp_{i}\leq z,\;i=1\ldots n$$

(20) $$\sum_{i=1}^{n}c_{i}^{k}=cs^{k},\;k=1\ldots K$$

(21) $$\left(x_{1,j}^{k}=1\right)\Rightarrow \left(fc_{j}^{k}=cs^{k}\right),j=1\ldots n;\;k=1\ldots K$$

(22) $$\left(x_{i,j}^{k}=1\right)\Rightarrow \left(fc_{j}^{k}=fc_{i}^{k}-c_{i}^{k}\right),i,j=1\ldots n;,\;k=1\ldots K$$

(23) $$\left(x_{1,j}^{k}=1\right)\Rightarrow \left(f_{1,j}^{k}=cs^{k}\right),j=1\ldots n;\;k=1\ldots K$$

(24) $$\left(x_{i,j}^{k}=1\right)\Rightarrow \left(f_{i,j}^{k}=fc_{j}^{k}\right),\;i,j=1\ldots n;\;k=1\ldots K$$

Energy consumption. The amount of energy needed to complete tasks performed by an UAV cannot exceed the maximum capacity of its battery.

(25) $$bat^{k}\leq CAP,\;k=1\ldots K$$

(26) $$\sum_{i=1}^{I}\sum_{j=1}^{I}x_{i,j}^{k}\times t_{i,j}\times P_{i,j}^{k}=bat^{k},\;k=1\ldots K$$

(27) $$P_{i,j}^{k}=\frac{1}{2}C_{D}\times A\times D\times {\left(va_{i,j}^{l}\right)}^{3}+\frac{{\left(ep+f_{i,j}^{k}\right)}^{2}}{D\times b^{2}\times va_{i,j}^{l}},$$

where $va_{i,j}^{l}$ and $t_{i,j}$ depend on the assumed strategies for goods delivering:

- Strategy 1—ground speed $vg_{i,j}^{l}$ is constant and airs peed $va_{i,j}^{l}$ is calculated from:
  (28) $$va_{i,j}^{l}=\sqrt{{\left(vg_{i,j}^{l}\times cos\vartheta_{i,j}-vw_{l}\times cos\theta_{l}\right)}^{2}+{\left(vg_{i,j}^{l}\times sin\vartheta_{i,j}-vw_{l}\times sin\theta_{l}\right)}^{2}}$$
  (29) $$t_{i,j}=\frac{d_{i,j}}{vg_{i,j}^{l}}$$
- Strategy 2—air speed $va_{i,j}^{l}$ is a constant and time $t_{i,j}$ is calculated due to Formula (29) where ground speed $vg_{i,j}^{l}$ is [45,47].
  (30) $$vg_{i,j}^{l}=\sqrt{{\left(va_{i,j}^{l}\times cos\varphi_{i,j}+vw_{l}\times cos\theta_{l}\right)}^{2}+{\left(va_{i,j}^{l}\times sin\varphi_{i,j}+vw_{l}\times sin\theta_{l}\right)}^{2}}$$
  (31) $$\varphi_{i,j}=\vartheta_{i,j}-arcsin\left(\frac{vw_{l}}{va_{i,j}^{l}}sin\left(\theta_{l}-\vartheta_{i,j}\right)\right)$$

Customer satisfaction. Customer satisfaction should be equal to or higher than CSL. Customer satisfaction is expressed by the following formula:

(32) $$\frac{\left(\sum_{i=1}^{n}pr_{i}\times cp_{i}\right)}{\left(\sum_{i=1}^{n}pr_{i}\times z_{i}\right)}\times 100\%\geq CSL$$

## 5. Problem Formulation

To find a solution to this type of problem, one has to answer the following question:

*Consider a UAVs fleet of size* K *servicing, in the lth flying time window (* $F_{l}$ *), customers belonging to the m-th cluster of the delivery distribution network (i.e., the subgraph* $CL_{m,l}$ *). Does there exist a set of sub-mission* $S_{m,l}$ *(determined by variables* Π,Y,C *) guaranteeing customers satisfaction* CSL *(31) under the constraints related to energy consumption (Formulae (25)–(31)), collision avoidance (Formula (13)), etc.?*

The investigated problem can be seen as a Constraint Satisfaction Problem (CSP) [18] given by Formula (33):

(33) CP=(V,D,C),

where:

V={Π,Y,C}—a set of decision variables determining sub-mission,
$S_{m,l}:\Pi$—a set of UAV routes,
Y—a schedule of a UAV fleet,
C—a set of payload weights delivered by the UAVs,
D—a finite set of decision variable domain descriptions,
C—a set of constraints specifying the relationships between UAV routes, UAV schedules, and transported materials Formulae (3)–(32).

To solve the *CP* in Formula (33), one has to determine the values of the decision variables for which all the constraints are satisfied. By implementing *C*P (Formulae (33)) in a constraint programming environment, such as IBM ILOG, one can answer the above formulated question.

## 6. Computational Experiments

**Figure 5:**
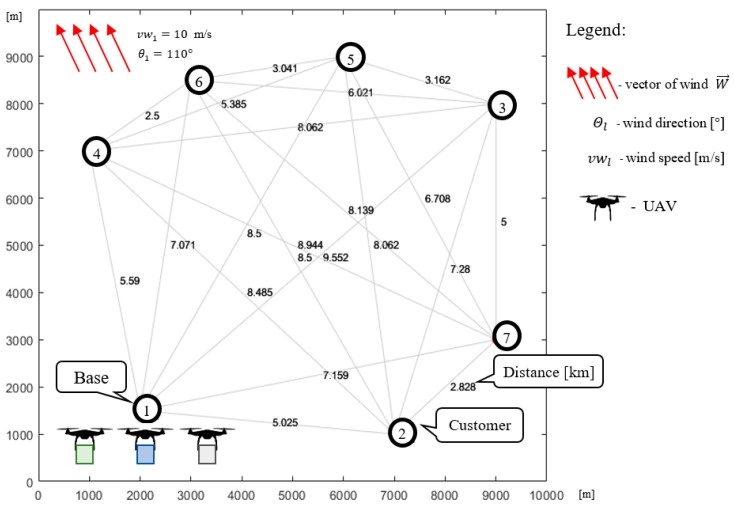
Transportation network from Cluster #1.

Consider the case shown in Figure 1, where the fleet consists of three homogeneous UAVs specified by technical parameters collected in Table 1. All customers’ demands (i.e., 30 kg for each node) should be satisfied within the time horizon 5000 s. Utilizing the proposed approach (Figure 5), the set of delivery points is subdivided into two clusters: Cluster #1 and Cluster # 2 (see Figure 1). The time horizon is divided into two flying time windows: $F_{1}=\left[0,\;2500\right]$ [s], $F_{2}=\left[2500,\;5000\right]$ [s]. For each time window, corresponding to periods of stable weather and arbitrarily selected delivery points, the corresponding sub-missions $S_{1,1}$ and $S_{2,2}$ are determined. Two kinds of weather conditions specified by vectors $\vec{W_{l}}=\left[vw_{l},\theta_{l}\right]$ are considered: $vw_{1}=10$ m/s, $\theta_{1}=110{}^{\circ}$ and $vw_{2}=12$ m/s, $\theta_{2}=150{}^{\circ}$. The problem under consideration can be reduced to seeking the answer to the following main question: *Does there exist flying mission composed from sub-missions* $S_{1,1}$ and $S_{2,2}$ *(determined by variables* Π,Y,C*), following the sequence of two flying time windows* while ensuring *100% customer satisfaction (*CSL=100%*) within the given time horizon?*

### 6.1. Cluster #1

In Cluster #1 (see Figure 5), covering an area of 100 km^2^, three UAVs deliver goods to six customers. Node $N_{1}$ represents the location of the company (i.e., the base from which the UAVs take off from/land) and nodes $N_{2}-N_{7}$ representing the locations of individual customers. Known is the demand of the individual customers for the goods transported by the UAVs, which is the same for each customer and equals 30 kg: $z_{1}=0$, $z_{2}=\ldots =z_{7}=30$. It is assumed that the UAVs must deliver to each customer the exact quantity of goods they demand.

The flying time window is equal to $F_{1}=\left[0,\;2500\right]$ [s]. The goods are transported under various weather conditions, which affect the rate of battery discharge; so, it is assumed that the wind speed is equal to $vw_{1}=10$ m/s and its direction is equal to $\theta_{1}=110{}^{\circ}$.

To answer the main question, the assumptions that describe delivery strategies are: (1) a constant ground speed and (2) a constant airspeed. In each case, the decreasing (along with the increasing length of distance traveled) UAV weight was taken into account. Appropriate formulations of the problem (Formula (33)) were implemented and solved in the declarative programming environment IBM ILOG (Intel Core i7-M4800MQ 2.7 GHz, 32 GB RAM).

**Figure 6:**
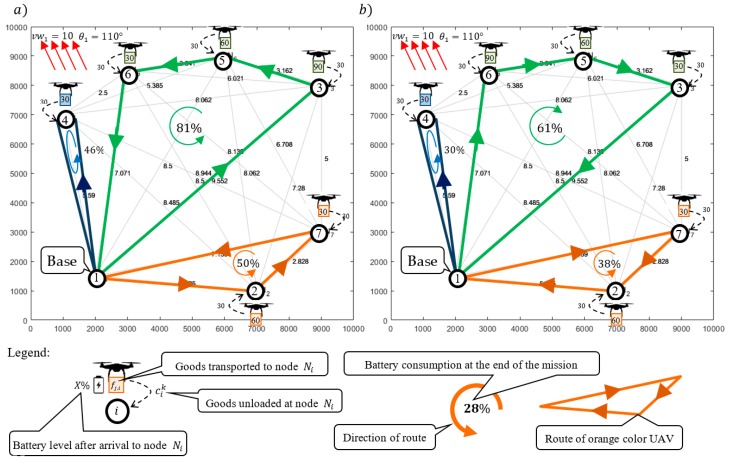
Obtained solution: (**a**) routes for Strategy 1, a constant ground speed; (**b**) routes for Strategy 2, a constant airspeed.

**Figure 7:**
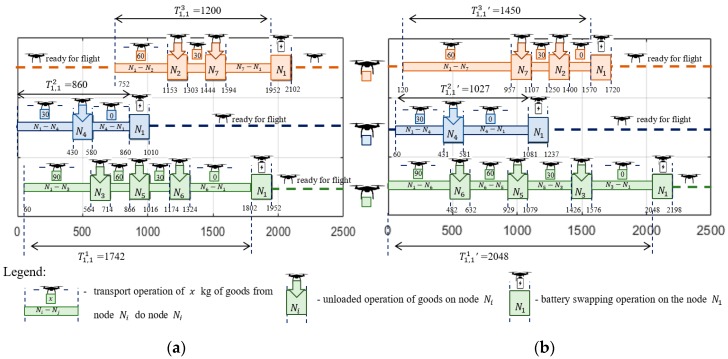
Obtained solution: (**a**) flight schedule for Strategy 1; (**b**) flight schedule for Strategy 2.

The solution providing sub-missions $S_{1,1}$ following Strategy 1 (i.e., constant ground speed) was obtained in 12.5 s. Figure 6a and Figure 7a show the computed flight routes and schedules. The obtained following routes $\pi_{1,1}^{1}=\left(N_{1},N_{3},N_{5},N_{6},N_{1}\right)$, $\pi_{1,1}^{2}=\left(N_{1},N_{4},N_{1}\right)$ and $\pi_{1,1}^{3}=\left(N_{1},N_{2},N_{7},N_{1}\right)$ guarantee that the required quantity of goods are delivered to customers.

As seen in Figure 7a, the corresponding flight times of individual UAVs participating in the sub-missions are, respectively: $T_{1,1}^{1}=1742$, $T_{1,1}^{2}=860$, $T_{1,1}^{3}=1200$. Customer satisfaction at all delivery points is 100% while the battery consumption of the UAVs travelling along routes $\pi_{1,1}^{1}$, $\pi_{1,1}^{2}$, and $\pi_{1,1}^{3}$ under given weather conditions is 81%, 46%, and 50%, respectively (battery capacity for each UAV is equal to: CAP=8000 kJ).

In turn, Figure 6b and Figure 7b show the computed sub-missions $S_{1,1}$′ (flight routes and schedules) following Strategy 2 (i.e., assuming constant airspeed). The obtained routes of UAVs are: $\pi_{1,1}^{1}\prime =\left(N_{1},N_{6},N_{5},N_{2},N_{1}\right)$, $\pi_{1,1}^{2}\prime =\left(N_{1},N_{4},N_{1}\right)$ and $\pi_{1,1}^{3}\prime =\left(N_{1},N_{7},N_{2},N_{1}\right)$. Similarly, as before, this solution guarantees that the required quantities of goods are delivered to customers under the given weather conditions. The missions shown in Figure 7a have corresponding flight times of for the individual UAVs of, respectively: $T_{1,1}^{1}\prime =2048$, $T_{1,1}^{2}\prime =1027$, and $T_{1,1}^{3}\prime =1450$.

Customer satisfaction at all delivery points is 100% while the battery consumption of the UAVs traveling along routes $\pi_{1,1}^{1}\prime$, $\pi_{1,1}^{2}\prime$, and $\pi_{1,1}^{3}\prime$ are, respectively, 61%, 30%, and 38% of CAP=8000 kJ. In comparison, the solution obtained with Strategy 2 is less energy-consuming than the solution obtained with Strategy 1. However, this comes at the cost of extended flight times of the UAVs participating in the sub-mission, i.e.: $T_{1,1}^{1}{}^{\prime }>T_{1,1}^{1}$, $T_{1,1}^{2}{}^{\prime }>T_{1,1}^{2}$, and $T_{1,1}^{3}{}^{\prime }>T_{1,1}^{3}$. The total flight time for Strategy 2 is 19% higher than is the case with Strategy 1.

**Figure 8:**
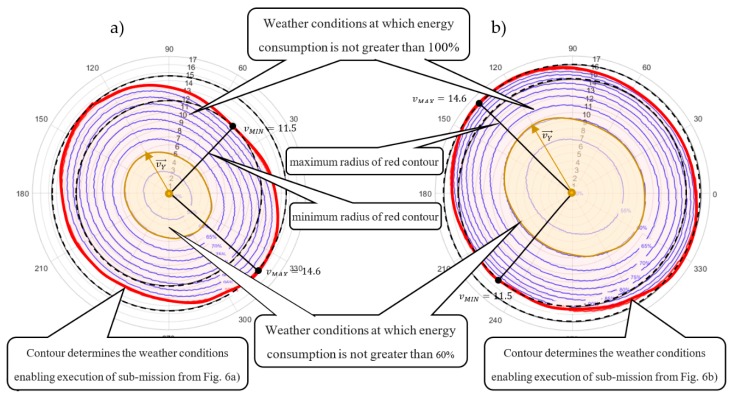
Radar charts of resistance to changes in wind speed: (**a**) Strategy 1; (**b**) Strategy 2.

Both solutions were analyzed in terms of sensitivity to the amount of energy consumption under various weather conditions. In the conducted analysis, it is assumed that the wind direction may change in the range from $\theta_{1}=0{}^{\circ}$ to $\theta_{1}=360{}^{\circ}$ and that the wind speed in a range from $vw_{1}=0$ to $vw_{1}=20$ m/s. Figure 8 shows radar charts illustrating the contour lines that determine the maximum value of the wind speed function (i.e., function parameterized by the wind direction) guaranteeing the fulfilment of all planned deliveries using the specified battery capacity limit in the range from 50% to 100% of CAP=8000 kJ. The contour lines connect the points of equal value of energy consumption. In that context, the blue contour lines determine the area of weather conditions for which execution of the sub-mission from Figure 6 can be fulfilled within the 50–100% range of the battery capacity limit CAP = 8000 kJ. In turn, the red contour line determines the weather conditions enabling the execution of feasible sub-missions from Figure 6. Crossing this line means that at least one of the UAVs exceeds its battery capacity limit.

In other words, the charts in Figure 8 illustrate how the obtained sub-missions are resistant to various weather conditions. For example, vector $\vec{v_{Y}}$ (distinguished inside the yellow area in Figure 8a) shows that the permissible (i.e., guaranteeing energy consumption less than 60% of initial value CAP=8000 kJ) speed of wind blowing at 120° for the sub-mission following Strategy 1 (i.e., assuming constant ground speed; see Figure 6a) is 5.9 m/s; but, in the case of the sub-mission following Strategy 2 (i.e., assuming constant airspeed; see Figure 6b) is 10 m/s. That means the sub-mission from Figure 6b following Strategy 2 is more resistant to changing weather conditions.

Radar charts indicate the wind speed $v_{MIN}$ (minimum radius of red contour line) at which the given deliveries can be completed regardless of the direction of the wind. The wind speed $v_{MIN}$ for the sub-missions from Figure 6a,b is $v_{MIN}=\;$11.5 and $v_{MIN}=\;$14.6 m/s, respectively. It is easy to see that the sub-mission of Figure 6b is the most robust to changing weather conditions, i.e., the permissible wind speed at which the execution of orders is guaranteed is 14.6 m/s. As already mentioned, increased resistance of such schedules is obtained at the expense of extending flight times leading to untimely delivery of planned deliveries.

Note that the contour lines of the second radar charts are spread out at different intervals. This means small changes in wind speed results in large changes in energy consumption. In that context, the sub-mission from Figure 6b is more resistant to changing weather conditions, than sub-mission from Figure 6a, but also more sensitive to their changes. The strategies highlight the tradeoffs encountered in mission planning between, timing, energy consumption, and equipment wear.

### 6.2. Cluster #2

**Figure 9:**
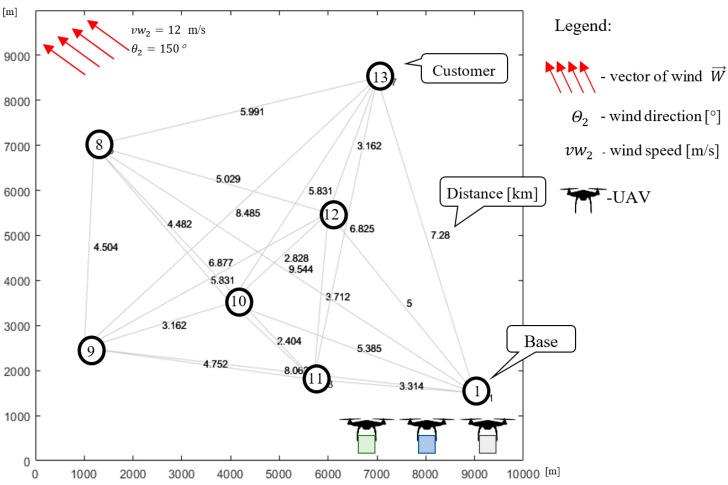
Transportation network from Cluster #2.

Let us consider Cluster #2 from Figure 9. As before, the UAVs deliver goods to six customers located in an area covering 100 km^2^. Nodes $N_{8}-N_{13}$ represent the locations of the individual customers. The demand of the individual customers is equal to: $z_{1}=0$, $z_{8}=\ldots =z_{13}=30$. The flying time window is equal to $F_{2}=\left[2500,\;5000\right]$ [s]. The weather conditions are changed, whereby the wind speed is higher, i.e., $vw_{2}=12$ m/s and the direction of wind is equal to $\theta_{2}=150{}^{\circ}$.

**Figure 10:**
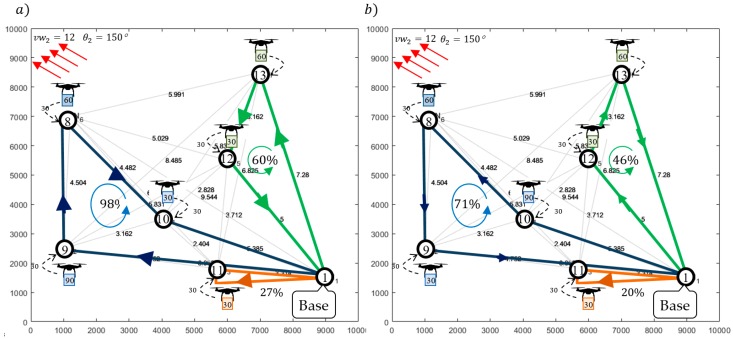
Obtained solution: (**a**) routes for Strategy 1; (**b**) routes for Strategy 2.

**Figure 11:**
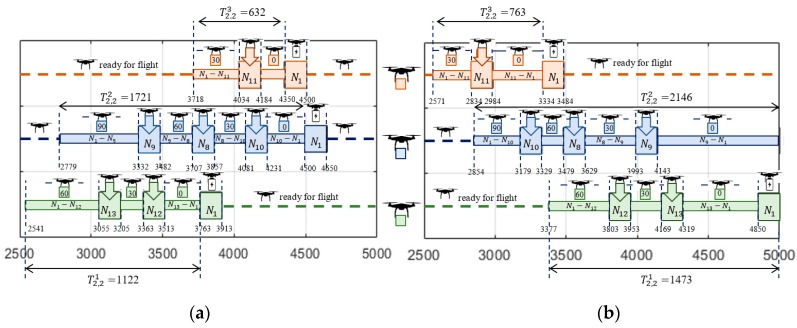
Obtained solution: (**a**) flight schedule for Strategy 1; (**b**) flight schedule for Strategy 2.

The solution providing sub-missions $S_{2,2}$ following Strategy 1 was obtained in 11.4 s by solving the problem (Formula (33)) in IBM ILOG. Figure 10a and Figure 11a show the computed routes and flight schedules. The obtained routes: $\pi_{2,2}^{1}=\left(N_{1},N_{13},N_{12},N_{1}\right)$, $\pi_{2,2}^{2}=\left(N_{1},N_{9},N_{8},N_{10},N_{1}\right)$, and $\pi_{2,2}^{3}=\left(N_{1},N_{11},N_{1}\right)$ guarantee that the required quantities of goods are delivered to customers under the given weather conditions i.e., $vw_{2}=12$ m/s and $\theta_{2}=150{}^{\circ}.$ Due to Figure 11a, the corresponding flight times of the UAVs participating in the sub-mission are, respectively, equal to: $T_{2,2}^{1}=1122$, $T_{2,2}^{2}=1721$, and $T_{2,2}^{3}=632$. Customer satisfaction at all delivery points is equal to 100%, while the battery consumption of the UAV traveling along routes $\pi_{2,2}^{1}$, $\pi_{2,2}^{2}$, and $\pi_{2,2}^{3}$ is at the level of 60%, 98%, and 27% of its initial capacity, respectively.

In turn, Figure 10b and Figure 11b show the computed sub-missions $S_{2,2}$ (routes and flight schedules) following Strategy 2, i.e., assuming constant airspeed. The obtained routes: $\pi_{2,2}^{1}\prime =\left(N_{1},N_{12},N_{13},N_{1}\right)$, $\pi_{2,2}^{2}\prime =\left(N_{1},N_{10},N_{8},N_{9},N_{1}\right)$, and $\pi_{2,2}^{3}\prime =\left(N_{1},N_{11},N_{1}\right)$ guarantee that the demanded quantity of goods are delivered to customers under the given weather conditions. As seen in Figure 11b, the corresponding flight times of individual drones participating in the sub-mission are: $T_{2,2}^{1}\prime =1473$, $T_{2,2}^{2}\prime =2146$, and $T_{2,2}^{3}\prime =763$, respectively. Customer satisfaction at all delivery points is equal to 100%, while the battery consumption of the UAVs traveling along these routes under the given weather conditions is 46%, 71%, and 20% of initial battery capacity.

**Figure 12:**
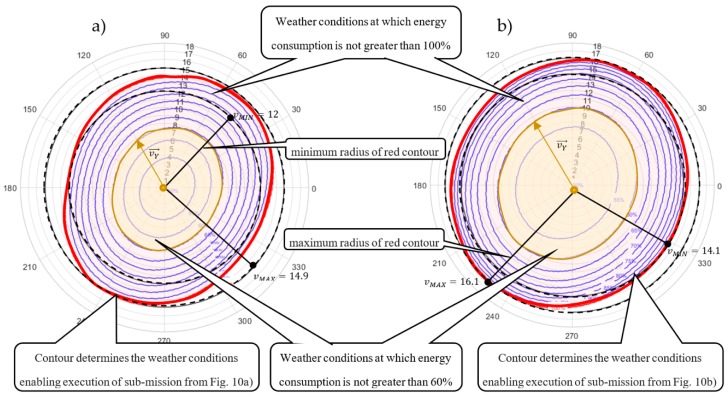
Radar charts of resistance to changes in wind speed: (**a**) Strategy 1; (**b**) Strategy 2.

The obtained solutions are analyzed in terms of energy consumption sensitivity for various weather conditions. It is assumed that the wind direction may change in the range from $\theta_{2}=0{}^{\circ}$ to $\theta_{2}=360{}^{\circ}$ and the wind speed may change in the range from $vw_{2}=0\;$ to $vw_{2}=20$ m/s. Figure 12 provides radar charts illustrating the contour lines (that can be treated as a function of wind direction), which determine the borders within which all planned sub-missions (shown in Figure 10) are fulfilled within the range from 50% to 100% of battery capacity limit.

In this case, vector $\vec{v_{Y}}$ (distinguished inside yellow area; see Figure 12a) determines the permissible (i.e., guaranteeing lees than 60% usage of battery capacity limit) speed of wind blowing at 120° for the sub-mission following Strategy 1 (Figure 10a), which is equal to 8 m/s and for the sub-mission following Strategy 2 (Figure 10b) is equal to 10 m/s.

Similarly, to the results obtained for Cluster #1, the contour lines of the second radar charts are not spread out at the same intervals. This means the sub-mission from Figure 10b is more resistant to weather changing conditions than sub-missions assuming a constant ground speed (see Figure 10a) though more sensitive to their changes.

### 6.3. Mission Planning

**Figure 13:**
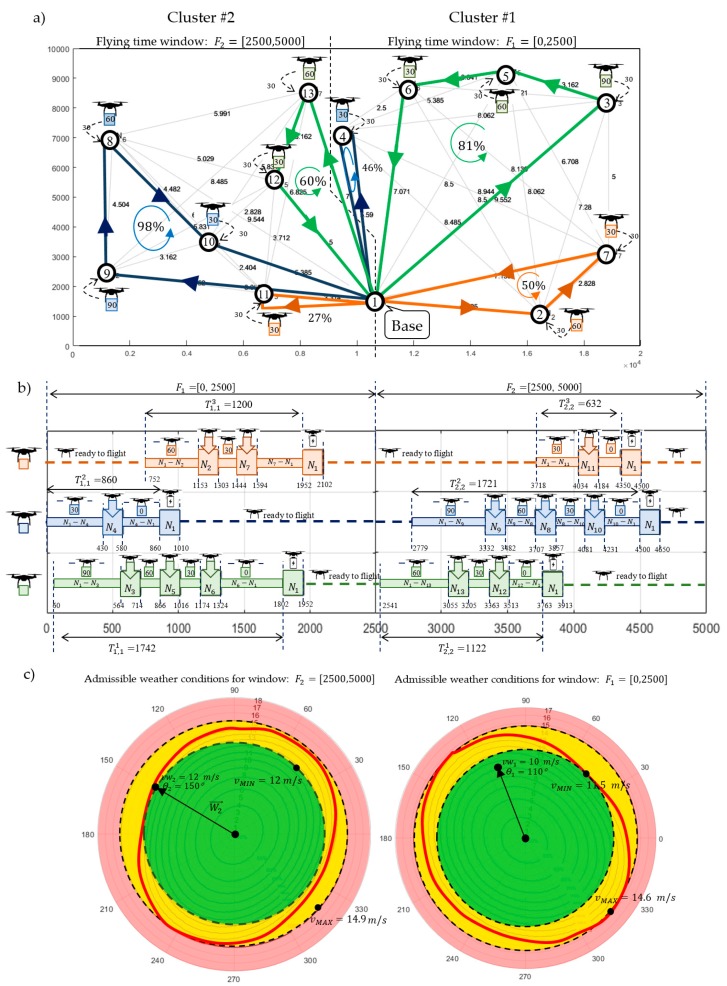
Example of the flying mission for the network from Figure 1.

The combined solutions obtained for Clusters #1 and #2 serve as a mission plan to serve the customers of the network in Figure 1. Figure 13 presents an example of the mission obtained from the sub-missions $S_{1,1}$ and $S_{2,2}$ presented in Figure 6a and Figure 10a. It should be noted that within the time window $F_{1}=\left(0,\;2500\right)$, goods are delivered to customers in Cluster #1: $N_{2}-N_{7}$. In turn, in time window $F_{2}=\left[2500,\;5000\right],$ goods are delivered to customers in Cluster #2: $N_{8}-N_{13}$. During the mission, none of the UAVs exceeds the permitted level of battery capacity. In Figure 13c, radar charts illustrating the zones of permitted weather conditions are presented. The green zone delimits the conditions under which the mission can be realized regardless of the wind direction. The size of the zone is determined by the value of $v_{MIN}$, which for Clusters #1 and #2 are 11.5 and 12 m/s, respectively. The orange zone, 11.5–14.6 and 12–14.9, defines weather conditions at which the mission is threatened during implementation, i.e., there exist conditions such as $vw_{2}=12$ m/s and $\theta_{2}=330$° at which UAVs exceed the set CAP limit. The red zone defines weather conditions ($vw_{1}$ > 14.6 and $vw_{2}$ > 14.9) at which the mission is not feasible due to excessive energy consumption. Figure 13c shows the vectors $\vec{W_{l}}=\left[vw_{l},\theta_{l}\right]$ describing the weather conditions considered in previous sections, i.e., $vw_{1}=10$ m/s, $\theta_{1}=110$°, and $vw_{2}=12$ m/s, $\theta_{2}=150$°.

Both vectors $\vec{W_{1}}=\left[10,\;110\right]$ and $\vec{W_{2}}=\left[12,\;150\right]$ are located in the green zone, which means that the occurrence of such conditions during the mission will not interrupt it. That means the obtained mission guarantees 100% customer satisfaction (delivery of all required goods to all customers) in the given horizon time (5000 s) under the given weather conditions.

### 6.4. Quantitative Results

In addition to the experiments reported above, we compared the effectiveness of the proposed model. The results of this analysis are shown in Table 2. The experiments included mission planning in distribution networks with four to ten nodes serviced by fleets consisting of four to ten homogeneous UAVs equipped with batteries capacity equal to CAP = 16,000 kJ while specified by parameters collected in Table 1.

The missions’ designation was carried out for three different weather conditions: $vw_{\;}=10$ m/s, $\theta_{\;}=30$°; $vw_{\;}=11$ m/s, $\theta_{\;}=130$°; and $vw_{\;}=12$ m/s, $\theta_{\;}=230$° following the two delivery strategies: Strategy 1, assuming a constant ground speed (i.e., $vg_{i,j}^{l}=20\;m/s)$; and Strategy 2, assuming a constant airspeed (i.e.,$\;va_{i,j}^{l}=20\;m/s$_)_. Experiments were conducted in the environment IBM ILOG (Intel Core i7-M4800MQ 2.7 GHz, 32 GB RAM).

The obtained results lead to the following observations:

- Interactive (i.e., online: t<300 s) support can be provided for networks consisting of no more than eight nodes. In practice, this means limiting decision making supported by DSSs to the distribution networks not exceeding eight nodes.
- An increase in the number of UAVs increases the route resistance (i.e., increasing of $v_{MIN}$ and $v_{MAX}$) to changes in weather conditions. For example, in a network of four nodes, the change from two to four UAVs increases value $v_{MIN}$ from 24.8 to 29.4 and $v_{MAX}\;$ from 28.1 to 33.9 for Strategy 1 (see yellow cells), as well as changing $v_{MIN}$ from 18.1 to 18.5 and $v_{MAX}$ from 19.0 to 19.2 for Strategy 2 (see green cells).
- The $v_{MIN}$ and $v_{MAX}$ values for route resistance in Strategy 2 are limited by the value of the airspeed ($va_{i,j}^{l}=20\;m/s$). This type of restriction does not exist in Strategy 1. This means that in situations where the wind speed exceeds the value *vw* > 20 m/s, it is recommended to use Strategy 1 (for this strategy, it is possible to get *v_MIN_* and $v_{MAX}$ above 20 m/s).

## 7. Conclusions

The declarative model proposed here (implemented in the ILGO IBM environment) allows one to determine UAV missions such that customer satisfaction levels are maximized under various weather conditions. The permissible size of the distribution network (12 nodes and 3 UAVs), for which such missions can be determined, makes the proposed model particularly suitable for application within an approach in which a network is decomposed into clusters, each covering a part of the set of all customers serviced during one flying time window. It is worth emphasizing that the possibility of taking into account the influence of weather conditions on energy consumption, and hence on the customer-servicing route and schedule, provides the basis for the construction of a model that allows identifying missions robust to specific weather changes.

As the considered UAVs routing problems are NP-hard, their solutions in real-life cases are only approximate. This means that approximate calculation techniques derived from artificial intelligence methods have to be used, especially employing a declarative representation following constraints programming paradigm. While the formulation of the problem is rather complex and not straightforward to simplify, the formulation arrived at in this research is validated in [4] and performed computer experiments. The experiments performed in the present study confirmed the efficiency of the proposed modeling concept implemented in UAV mission planning. Two policies aimed at minimizing total travel time at the cost of saving battery power were considered. Special attention was paid to research focused on the sensitivity of energy consumption due to wind speed and direction changes. Real-life implementations of these types of systems tend to be exceedingly complex due to the very nature of the complex decision problem addressed and the cost of operating UAVs in this class. Future work will focus on reducing the complexity of the formulation. However, in the current work, the focus is on validating that several strategies are viable and that it is feasible to create and evaluate such alternatives for realistic scale problem instances.

In our future research on resistant UAV mission planning, we plan to explore the relationships linking the total distance traveled with the total travel time and the cost of saving the battery power of a UAV fleet. Particular attention will be paid to the pick-up delivery problem with time windows and to planning the size of fleets composed of heterogeneous UAVs. Efforts aimed at practical verification of the results obtained, conditioned by the authorities allowing access to areas where Beyond Visual Line of Sight aircrafts could be tested will play a pivotal role.

## Figures and Tables

**Table 1:** Technical parameters.

Technical Parameters of UAVs	Value	Unit
Payload capacity (Q)	90	kg
Battery capacity (CAP)	8000	kJ
Airspeed (va)	20	m/s
Drag coefficient ($C_{D}$)	0.54	-
Front surface of UAV (A)	1.2	m
UAV width (b)	8.7	m

**Table 2:** Results of selected experiments.

n ^1)^	K	Assumptions	$v_{w}=10\;m/s$				$v_{w}=11\;m/s$				$v_{w}=12\;m/s$				*NDV*	*NC*
			Θ=30°				Θ=130°				Θ=230°					
			E	TC	$v_{MIN}$	$v_{MAX}$	E	TC	$v_{MIN}$	$v_{MAX}$	E	TC	$v_{MIN}$	$v_{MAX}$		
4	2	Strategy 1	29.80	3.73	24.8	28.1	19.17	3.71	24.6	28.2	29.8	3.74	24.8	28.1	828	356
		Strategy 2	19.1	3.82	18.1	19.0	19.18	3.78	18.1	19.1	19.1	3.76	18.1	19.0	828	356
	3	Strategy 1	13.59	3.89	29.4	33.9	13.59	3.97	29.4	33.9	13.59	3.95	29.4	33.9	1774	654
		Strategy 2	13.57	3.75	18.5	19.2	13.57	3.96	18.5	19.2	13.57	3.81	18.5	19.2	1774	654
	4	Strategy 1	13.59	4.24	29.4	33.9	13.59	4.17	29.4	33.9	13.59	4.35	29.4	33.9	3076	1036
		Strategy 2	13.57	4.31	18.5	19.2	13.59	4.28	18.5	19.2	13.57	4.36	18.5	19.2	3076	1036
6	2	Strategy 1	35.67	4.44	23.6	25.6	22.62	4.23	23.6	25.6	22.62	4.60	23.6	25.6	3014	910
		Strategy 2	22.59	4.31	17.9	18.6	22.59	4.38	17.9	18.6	22.81	4.12	17.9	18.6	3014	910
	3	Strategy 1	19.4	7.12	25.8	27.7	19.40	5.25	25.8	27.7	19.4	8.08	25.8	27.7	7476	1902
		Strategy 2	19.37	6.34	18.2	18.7	19.38	5.24	18.2	18.7	19.37	9.32	18.2	18.7	7476	1902
	4	Strategy 1	19.4	9.98	25.8	27.7	19.40	6.44	25.8	27.7	19.4	13.67	25.8	27.7	13,910	3528
		Strategy 2	19.37	8.19	18.2	18.7	19.38	9.46	18.2	18.7	19.37	8.08	18.2	18.7	13,910	3528
8	2	Strategy 1	22.62	46.04	20.9	25.6	24.21	7.93	18.8	25.1	22.62	8.63	23.6	25.6	9552	2248
		Strategy 2	22.59	102.93	17.9	18.6	24.18	281.67	17.7	18.6	22.59	9.43	17.9	18.6	9552	2248
	3	Strategy 1	22.62	t > 300	23.6	25.6	20.62	19.94	25.3	26.9	25.15	231.63	18.8	24.2	25,898	5358
		Strategy 2	25.13	59.49	17.8	18.2	23.79	71.73	17.8	18.6	25.13	126.89	17.8	18.2	25,898	5358
	4	Strategy 1	22.55	t > 300	23.6	25.6	20.62	128.00	25.3	26.9	25.15	105.18	18.8	24.2	49,960	9800
		Strategy 2	25.13	110.94	17.8	18.2	21.33	95.87	18.0	18.7	25.49	115.29	17.7	18.2	49,960	9800
10	2	Strategy 1	27.54	t > 300	18.9	22.9	28.59	183.53	18.2	22.8	29.46	t > 300	17.8	21.7	21,402	4530
		Strategy 2	24.18	t > 300	17.9	18.4	25.65	t > 300	17.5	18.0	29.35	t > 300	19.5	20.6	21,402	4530
	3	Strategy 1	28.38	t > 300	18.7	22.5	23.83	t > 300	20.0	25.1	24.29	t > 300	18.9	25.3	59,920	11,502
		Strategy 2	24.23	t > 300	17.5	18.5	24.23	t > 300	17.5	18.5	24.23	t > 300	17.5	18.5	59,920	11,502
	4	Strategy 1	26.21	t > 300	19.5	23.2	24.29	t > 300	18.9	25.3	26.2	t > 300	18.4	24.3	116,986	21,622
		Strategy 2	26.18	t > 300	17.6	18.3	26.19	t > 300	17.3	18.4	26.19	t > 300	17.3	18.4	116,986	21,622

n^1)^—number of nodes; K—size of the UAV fleet; E—maximum consumed energy (%); TC—time of computation (s); NC—number of constraints; NDV—number of decision variables
